# External Laryngocele in an Adult: A Case Report

**DOI:** 10.7759/cureus.73234

**Published:** 2024-11-07

**Authors:** Surintheren Kumar Tamilchelvan, Atikah Rozhan, Sharmini Kuppusamy, Zubaidah Hamid

**Affiliations:** 1 Otolaryngology, International Islamic University Malaysia, Kuantan, MYS; 2 Otorhinolaryngology - Head and Neck Surgery, International Islamic University Malaysia, Kuantan, MYS; 3 Otolaryngology - Head and Neck Surgery, Hospital Tuanku Ampuan Najihah, Kuala Pilah, MYS

**Keywords:** laryngocele, orl-hns, rare head and neck, swelling of neck, valsalva maneuvers

## Abstract

Laryngocele is a rare condition marked by an abnormal enlargement of the air-filled saccule of the laryngeal ventricle. This case report showcases a distinctive presentation of external laryngocele to assist clinicians in its diagnosis and management. A 43-year-old male, with a 20-year history of painless swelling on the right side of his neck, likened to the size of an orange, presented with a recent increment in size. He noticed a gurgling sound when pressing on the swelling but did not experience any hoarseness or difficulty swallowing. During the physical examination, it was observed that there was a swelling on the right side of the neck at level II that measured approximately 3 x 5 cm. This swelling seemed to increase when the Valsalva maneuver was performed. A computed tomography scan revealed a 5 x 3 cm air-filled lesion, indicative of an external laryngocele. Although surgical excision was advised, the patient decided not to proceed with treatment and did not attend follow-up appointments. Laryngocele mainly impacts men, especially those in their fifth and sixth decades of life and is linked to activities that raise laryngeal pressure. Diagnosis is mainly based on clinical evaluation, complemented by imaging techniques such as CT and MRI. Surgical excision remains the preferred treatment, with approaches differing, depending on the laryngocele subtype. This particular case highlights the infrequency of laryngocele, and how it may manifest as a swelling in the neck. It underscores the importance of clinicians being aware of this harmless condition, highlighting the significance of taking a detailed patient history and using suitable imaging for accurate diagnosis and effective management, especially to rule out any malignancies. This report adds to the current body of knowledge on laryngocele, offering valuable information on its clinical symptoms and treatment implications.

## Introduction

A laryngocele is an uncommon ailment that develops from an irregular enlargement of the air-filled saccule of the laryngeal ventricle [1-3]. It creates an air sac that is covered with pseudo-stratified ciliated columnar epithelium and remains connected to the ventricle through a narrow stalk [1]. There are three types of laryngoceles: internal, external, and combined or mixed laryngocele, based on their connections with the thyrohyoid membrane. Laryngocele affects one in 2.5 million people worldwide [3]. The differential diagnosis for laryngocele encompasses a saccular cyst, branchial cyst, abscess in the neck, and lymphadenopathy. We would like to highlight this external laryngocele case due to its rarity and to help clinicians with accurate diagnosis and management.

## Case presentation

A 43-year-old gentleman with no known comorbidities and a smoker presented with painless right neck swelling for the past 20 years. Further history revealed that the size was about that of an orange, and it has slightly increased in size over the past year. He claims whenever he applies pressure to the swelling, it produces a gurgling sound, which is a concern for him. He denies any hoarseness, odynophagia, or dysphagia. The presence of any nasal symptoms was refuted by the patient. His appetite is good, and there has been no weight loss. By profession, he is attached to the maintenance section of the electrical board and occasionally lifts heavy tools. His activities of daily living are not affected by this swelling, and there is no disturbance in his respiration. The patient affirms no family history of malignancy. The patient is the sole breadwinner and currently lives with his wife and three children.

**Figure 1:**
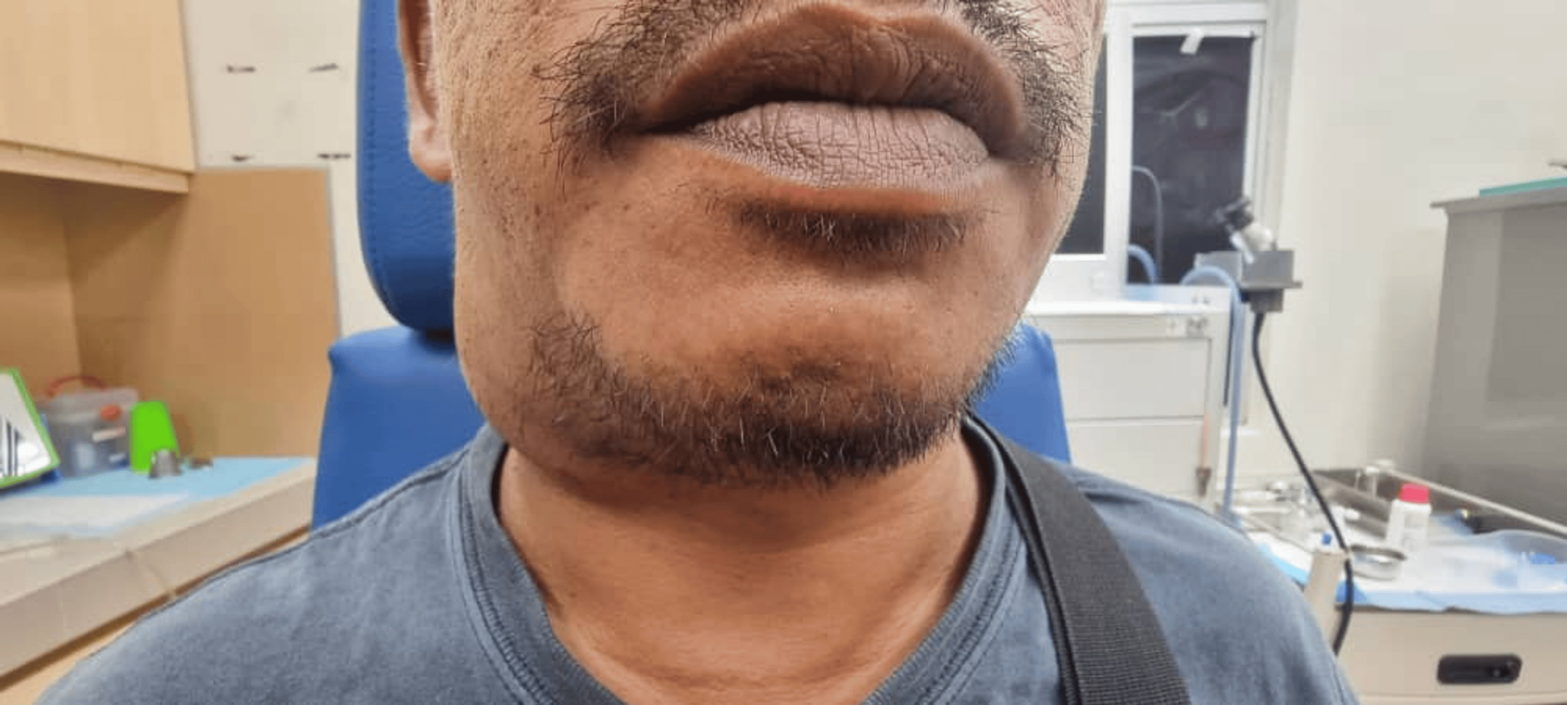
Right-sided level II swelling

**Figure 2:**
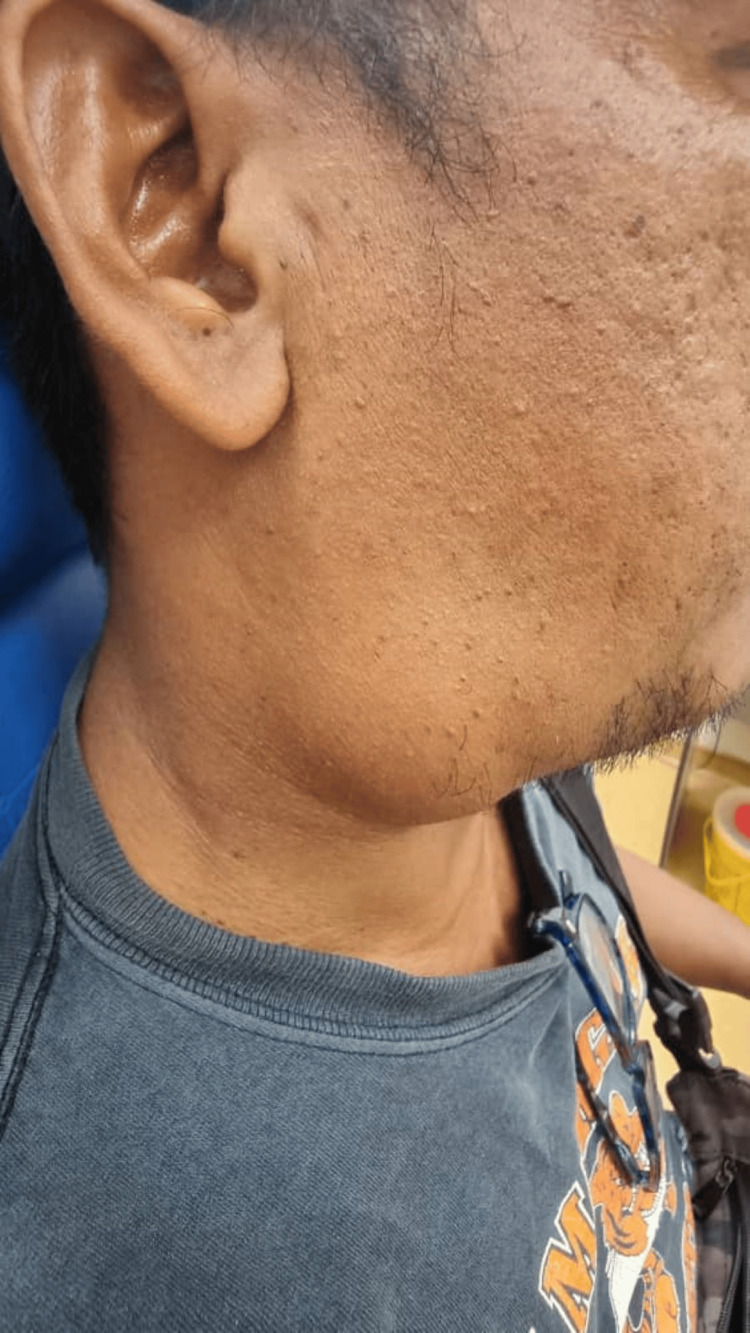
Borders of the swelling are not well delineated. The swelling increases in size with Valsalva maneuver.

The patient is of moderate build, comfortable under room air, and not in respiratory distress. Neck examination revealed a right-sided level II swelling measuring about 3 x 5 cm (Figure 1). The swelling is soft in nature with no skin changes, and the borders are not well-delineated (Figure 2). The swelling increases in size with the Valsalva maneuver and, upon compression, produces a hissing sound. Flexible fiberoptic nasopharyngolaryngoscopy was unremarkable.

**Figure 3:**
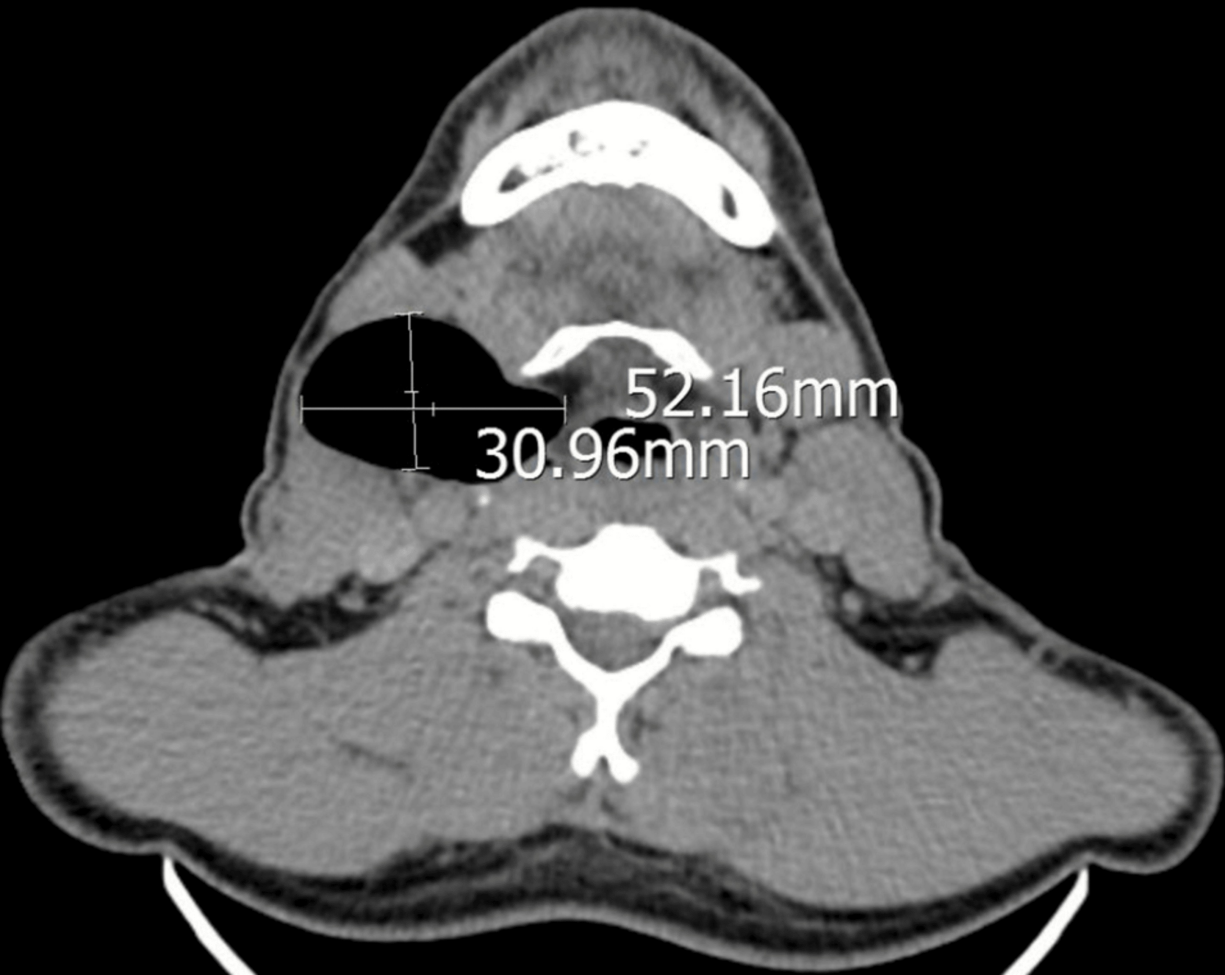
A 5 x 3 cm air-filled lesion between the right submandibular and right carotid space.

**Figure 4:**
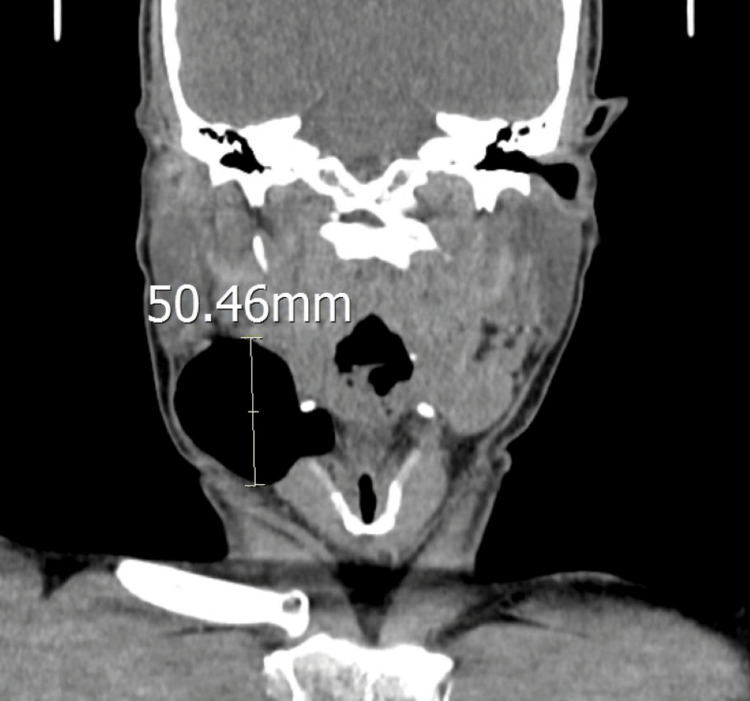
Lesion extends inferiorly to the level of the arytenoids. Airway is patent.

We proceeded with a computed tomography scan of the neck, which revealed a 5 x 3 cm air-filled lesion between the right submandibular and right carotid space (Figure 3). It extends inferiorly to the level of the arytenoids (Figure 4). A diagnosis of external laryngocele was made. The patient was advised to undergo surgical excision; however, he was not keen on it. Subsequently, the patient defaulted on our follow-up.

## Discussion

Laryngocele can be challenging to many due to its scarcity. Inherited or acquired etiology can be used to explain how laryngocele develops [1,2]. It is predominantly more common in males, with a sevenfold higher incidence compared to females, peaking in the fifth and sixth decades of life. Seventy-five percent of laryngocele cases have been discovered to be single-sided, showing no preference for the right or left side [1].

Dysphonia or swelling in the neck, which usually becomes more noticeable during the Valsalva maneuver, are common symptoms of laryngoceles. Cough, dyspnea, inspiratory stridor, dysphagia, and a feeling of a foreign body in the throat are some additional symptoms [2]. Laryngocele may be seen in trumpet players and glassblowers who work with elevated intraluminal laryngeal pressure for extended periods. Moreover, laryngeal tumors that cause mechanical blockage raise intralaryngeal pressure, leading to laryngocele. Supraglottic carcinoma is the most common laryngeal carcinoma reported to be associated with laryngocele. It may result in a valve-like closure at the neck of the ventricular appendage, allowing the entrance of air but preventing its exit. Conditions, such as scleroderma and systemic lupus erythematosus, may also contribute to this condition [3].

The majority of authors divide laryngocele into three categories: internal (contained within the thyrohyoid membrane), external (penetrating superiorly through the thyrohyoid membrane into the subcutaneous tissues of the neck), and combined (mixture of internal and external) [4]. Mixed laryngocele is a term used when both conditions are present. According to research, mixed laryngocele is the most prevalent (44%), followed by internal (30%) and external (26%) [5].

Laryngocele is primarily diagnosed clinically. Plain radiographs of the soft tissue of the neck are helpful, particularly if the Valsalva maneuver is used [1,6]. A computed tomography scan offers a conclusive diagnosis through cross-sectional imaging. Magnetic resonance imaging provides precise information about the laryngocele's borders and its relationship to the thyrohyoid membrane [7]. The preferred imaging method for laryngocele is magnetic resonance imaging, as it helps to differentiate between neoplastic illness and blocked mucus and inflammation [8].

The treatment of choice is surgical excision [1,7,8]. The three different types of laryngoceles necessitate multiple approaches for its excision [1,7,8]. Surgeries are performed via the external approach, mainly for external and combined laryngocele, and the endolaryngeal approach, mainly for internal laryngocele. An external method offers great visibility while examining the boundary between the laryngocele's neck and the adjacent paraglottic tissue. Moreover, this method leads to a lower chance of recurrence, minimal side effects, and almost no complications. Endoscopic removal using a CO_2_ laser is the preferred option for those with internal laryngocele. This technique takes less time for the procedure and results in minimal harm to the larynx and vocal cords. The voice and swallowing abilities can be maintained [1,8].

## Conclusions

This case report underscores the rarity and clinical importance of laryngocele, specifically focusing on its external subtype. The case presented is of a 43-year-old male with a persistent, painless swelling in the neck. It showcases the distinctive characteristics and difficulties linked with this particular condition. Clinicians need to keep a vigilant eye out for laryngocele during neck swelling assessments, particularly in individuals with professional backgrounds that could heighten laryngeal pressure. A comprehensive evaluation, which involves a detailed patient history and relevant imaging, is essential for distinguishing laryngocele from potentially more severe conditions, such as malignancies. Surgical excision is typically recommended as the preferred treatment, but it is essential to consider the patient's preferences and their adherence to follow-up care. By sharing this case, we aim to improve the awareness and understanding of laryngocele, thus aiding in its improved recognition and management in clinical practice.
